# SMYD2 inhibitors have no effect in improving non-alcoholic steatohepatitis in mice

**DOI:** 10.3389/fendo.2025.1480453

**Published:** 2025-06-05

**Authors:** Lanzexin Yang, Shixuan Zhuo, Xinyu Zhu, Xinhui Zhang, Zinan Wang, Yan Chen

**Affiliations:** ^1^ Shanghai Institute of Nutrition and Health, University of Chinese Academy of Sciences, Chinese Academy of Sciences, Shanghai, China; ^2^ School of Clinical Medicine, Gannan Medical University, Ganzhou, Jiangxi, China

**Keywords:** SMYD2, non-alcoholic steatohepatitis, liver injury, fibrosis, inflammation, hepatic steatosis

## Abstract

**Introduction:**

Nonalcoholic steatohepatitis (NASH), characterized by progressive liver injury, inflammation, and fibrosis, is a leading chronic liver disease worldwide. Pharmacotherapy for NASH is thus urgently needed. Through a strategy of *in vivo* lineage tracing, it was recently discovered that deletion of a protein methyltransferase SMYD2 has a protective role in hepatic steatosis. In this study, we evaluated the potential therapeutic effect of two SMYD2 inhibitors AZ505 and LLY-507 in a mouse NASH model.

**Methods:**

The mouse NASH model was induced by a choline-deficient, L-amino acid-defined, high-fat diet (CDAHFD) for 12 weeks. SMYD2 inhibitors AZ505 and LLY-507 were administered in the last 4 weeks at a dose of 10 mg/kg by intraperitoneal injection three times per week. A series of biochemical and histological analyses were conducted to determine the therapeutic potential of SMYD2 inhibitors.

**Results:**

The inhibitory effect of AZ505 and LLY-507 on histone methylation was confirmed with liver samples. CDAHFD was able to induce marked liver fibrosis and inflammation in the mice. However, treatment of the mice with AZ505 and LLY-507 failed to show any improvement in NASH scores, liver damage, liver fibrosis, macrophage infiltration, or hepatic inflammation in mice.

**Discussion:**

In conclusion, our findings suggest that SMYD2 inhibition is not an effective strategy to alleviate NASH at least in mice.

## Introduction

The overall prevalence of non-alcoholic fatty liver disease (NAFLD) worldwide is estimated to be 30% and increasing, which presents a substantial global health challenge (1–3). NAFLD represents a spectrum of liver disorders associated with metabolic syndrome ranging from simple fatty liver to more severe nonalcoholic steatohepatitis (NASH) (4). The transition from relatively benign hepatic steatosis to NASH marks a critical step in NAFLD progression that has important clinical implications during which metabolic dysregulation, inflammation responses, and fibrosis are closely intertwined (5, 6). For the majority of at-risk patients, including those with NAFLD, hepatic steatosis serves as an important prognostic indicator of cardiovascular and cerebrovascular risk. Concurrently, inflammatory processes and hepatocyte injury drive disease progression toward NASH and subsequent fibrosis. This fibrotic progression is particularly clinically significant, as the stage of fibrosis is predictive of liver-specific morbidity and mortality. Ultimately, patients with NASH and advanced fibrosis may develop end-stage complications, including cirrhosis, portal hypertension, or hepatocellular carcinoma (HCC), all of which are associated with substantially worse prognosis (7–11). Although there are no approved therapies for the treatment of NASH, progress in the understanding of its pathogenesis has resulted in the identification of many pharmacological targets. Numerous drugs are currently undergoing phases 2 and 3 clinical trials focusing on various mechanisms of action (12, 13).

Recent advancements in research have identified novel targets for NAFLD using an innovative *in vivo* genetic lineage tracing strategy to identify genes that affect liver clonal expansion (14). It was found that SET and MYND domain-containing protein 2 (SMYD2) is one of the candidate genes whose deletion or inhibition has a protective role against lipotoxicity in a mouse model fed with Western diet (14). SMYD2 is a protein methyltransferase that methylates histone H3 at lysine 4 (H3K4) or lysine 36 (H3K36), as well as diverse nonhistone proteins (15–17). Abnormal expression or dysfunction of SMYD2 is implicated in various diseases underscoring its potential as a promising target for diseases such as cardiovascular disease and cancer (18). In this study, we aimed to determine whether SMYD2 inhibitors could ameliorate NASH development in mice.

## Materials and methods

### Animals

Male C57BL/6 mice at 8 weeks of age were purchased from Shanghai Laboratory Animal Co., Ltd. (Shanghai, China). To induce NASH, mice were fed with a choline-deficient, L-amino acid-defined, high-fat diet (CDAHFD) (A06071302, Research Diets) for 12 weeks. CDAHFD has been widely used to establish a preclinical model that mimics human NASH features including steatosis, inflammation, and pericellular fibrosis (19–21). The vehicle (10% DMSO in PBS) or SMYD2 inhibitors (10 mg/kg) were administered by intraperitoneal injection three times per week during the last 4 weeks as previously reported (14). All mice were housed under a 12:12-h light/dark cycle at a controlled temperature. All mice were anesthetized with 2%–3% isoflurane inhaled for 2–3 min and then sacrificed by rapid cervical dislocation to avoid unnecessary pain and suffering before death. All animal experimental protocols were approved by the Institutional Animal Care and Use Committee at the Shanghai Institute of Nutrition and Health, Chinese Academy of Sciences, Shanghai, China, with an approval number SINH-2024-CY-1 (approval data: 30 May 2024).

### Reagents

AZ505 and LLY-507 were purchased from MCE (NJ, U.S.A.). The TRIzol reagent was from Invitrogen (Carlsbad, CA, U.S.A.). RIPA buffer was from Yeasen (Shanghai, China). Antibodies against Tri-Methyl-Histone H3 (Lys4) (C42D8) (Catalog No. 9751S) were from Cell Signaling Technology (Boston, MA, U.S.A.); antibodies against Histone H3 (Catalog No. A22348) and GAPDH (Catalog No. AC033) were from ABclonal (Wuhan, Hubei, China).

### Sample preparation

Blood was collected upon euthanasia. Approximately 100 mg of frozen liver tissue was extracted in 1 ml of chloroform:methanol (2:1 v/v). Samples were rotated after 4–6 h and centrifuged at 620 rcf (*g*) for 10 min at 4°C. Supernatant was collected and placed in a fume cupboard overnight. Samples were dissolved in ethanol containing 1% Triton X-100 for subsequent measurements. Blood levels of aspartate aminotransferase (AST) and alanine aminotransferase (ALT) were measured using assay kits from ShenSuoYouFu (Shanghai, China). The concentration of liver TG was normalized to tissue weight.

### Hematoxylin and eosin, Sirius Red staining, and immunohistochemistry

Livers were isolated from mice and fixed overnight with 4% paraformaldehyde (PFA) for paraffin embedding. Sectioning, H&E, and Sirius Red staining, and immunostaining for CD11b and F4/80 were performed by Servicebio (Wuhan, Hubei, China). Immunostaining for α-SMA was performed by Pinuofei (Wuhan, Hubei, China). The images of the slides were captured using an Olympus BX51 microscope.

### Western blotting

Liver samples were lysed in RIPA buffer with fresh protease inhibitors (MCE) and phosphatase inhibitors (Sigma-Aldrich), and the supernatant was collected after centrifugation at 13,200 rcf (*g*) for 10 min at 4°C. Total lysate protein levels were quantified using a BCA Protein Assay kit (Beyotime) according to the manufacturer’s protocols. Proteins were fractionated using 15% SDS-PAGE gels and transferred to PVDF (Fisher Scientific) membranes. Membranes were probed with primary antibody at 4°C overnight. After incubation with secondary antibody conjugated to HRP, the membranes were scanned using Tanon-5200. The dilution ratio of the primary antibodies and secondary antibodies were 1:1,000 and 1:5,000, respectively. Quantitative analysis of the Western blotting bands was performed using ImageJ software.

### Real-time quantitative PCR analysis

Total RNA of livers was extracted using TRIzol reagent. cDNA was acquired by reverse transcription using FastQuant RT Kit (Tiangen, Shanghai, China). Real-time quantitative PCR was performed using SYBR Green PCR system (TOYOBO, Tokyo, Japan) with specific primers (**Supplementary Table S1**). The PCR reactions were performed with an ABI QuantStudio6 system. The mRNA levels of target gene expression were normalized to the average value of β-actin.

### Statistical analysis

Data were expressed as mean ± SEM. Statistical significance was evaluated using one-way ANOVA analysis for more than two groups. Differences were considered significant at a p-value < 0.05.

## Results and discussion

**Figure 1:**
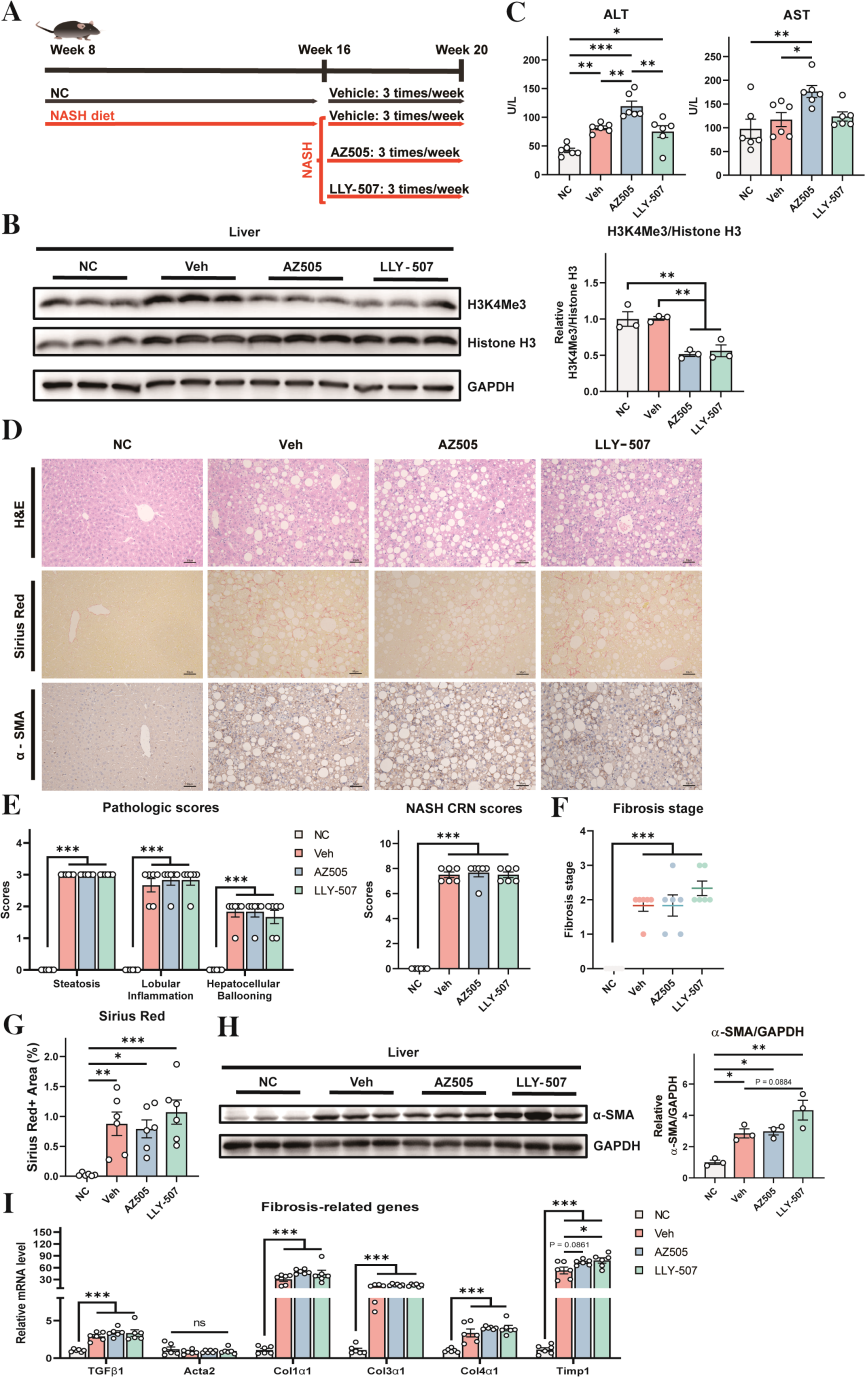
Effects of SMYD2 inhibitors on liver injury and fibrosis in a mouse NASH model. **(A)** A schematic diagram of the experimental strategy (n = 6 for each group). Eight-week-old mice were fed with normal chow (NC) for 12 weeks (as a negative control) or with CDAHFD for 12 weeks to induce NASH. The NASH groups were treated with vehicle control (10% DMSO in PBS, as a positive control) or SMYD2 inhibitors AZ505 or LLY-507 (10 mg/kg) by intraperitoneal injection three times per week in the last 4 weeks. **(B)** Western blotting of liver proteins to validate the inhibitory effect of AZ505/LLY-507 in decreasing trimethylation of histone H3 at lysine 4 (H3K4). Quantification of the result is shown in the right pane. **(C)** Serum ALT and AST levels of the mice (n = 6). **(D)** Representative H&E staining, Sirius Red staining, and immunostaining for α-SMA in the liver sections of mice (scale bars: 50 μm). **(E, F)** Pathologic scores, NASH CRN scores, and fibrosis stage scores based on H&E staining results (n = 6). The CRN scores are the sum of steatosis, hepatocellular ballooning, and lobular inflammation scores. **(G)** Quantification of the Sirius Red-positive area (n = 6). **(H)** Western blotting to detect α-SMA protein level in the liver of the mice. Quantitation of the result is shown in the right panel. **(I)** The mRNA levels of fibrosis-related genes in the liver (n = 6). All statistical data are shown as mean ± SEM. *p < 0.05, **p < 0.01, ***p < 0.001, ns for non-significant.

To investigate the role of SMYD2 inhibitors in NASH, a diet-induced NASH model was generated in mice using a choline-deficient, L-amino acid-defined, high-fat diet (CDAHFD) (**Figure 1A**). Eight-week-old male WT C57BL/6J mice were divided into four groups. The first group was a negative control that received normal chow diet for the entire period of 12 weeks (**Figure 1A**). The other three groups were fed with CDAHFD for 12 weeks. The positive control group was treated with vehicle control (10% DMSO in PBS) in the last 4 weeks. The drug treatment groups were treated with SMYD2 inhibitors AZ505 or LLY-507 (22, 23) in the last 4 weeks (**Figure 1A**). The dose and usage of the inhibitors were identical to the protocol as previously reported (14). Compared to the vehicle control group, mice treated with either SMYD2 inhibitors showed a significant reduction in histone H3 trimethylation at lysine 4 (H3K4) in the liver, thus validating the inhibitory effect of AZ505 or LLY-507 on the enzymatic activity of SMYD2 (**Figure 1B**). Serum levels of alanine aminotransferase (ALT) and aspartate aminotransferase (AST) were elevated in all the NASH mice confirming that CDAHFD could induce liver damage of the mice. Both ALT and AST were further increased in the AZ505-treated group suggesting exacerbation of hepatocyte damage by AZ505 treatment (**Figure 1C**).

Histological examination revealed a clear development of NASH by CDAHFD (**Figures 1D–F**, the vehicle group compared with the NC group). The NASH CRN system depicts the nonalcoholic fatty liver disease activity score (NAS), which is a composite score of steatosis, lobular inflammation, hepatocellular ballooning, and fibrosis (disease stage) (24, 25). However, treatment with SMYD2 inhibitors could not improve NASH pathology in the liver (**Figures 1D–F**). Histological analysis and Sirus Red staining also manifested apparent hepatic fibrosis by CDAHFD, while SMYD2 inhibitor had no improvement on fibrosis in the liver (**Figures 1D, F, G**). Histochemical staining and Western blotting with α-SMA further revealed that the development of fibrosis occurred in the liver after treatment with CDAHFD, while SMYD2 inhibitors could not reverse it (**Figures 1D, H**). We also analyzed the expression levels of a series of fibrosis-related genes in the liver (26, 27), and found that CHAHFD-induced expressions of these genes could not be reversed by SMYD2 inhibitors (**Figure 1I**). Collectively, these data indicated that CDAHFD could induce overt development of histological features of NASH and fibrosis in the liver, while these NASH features could not be improved by SMYD2 inhibitors AZ505 and LLY-507.

**Figure 2:**
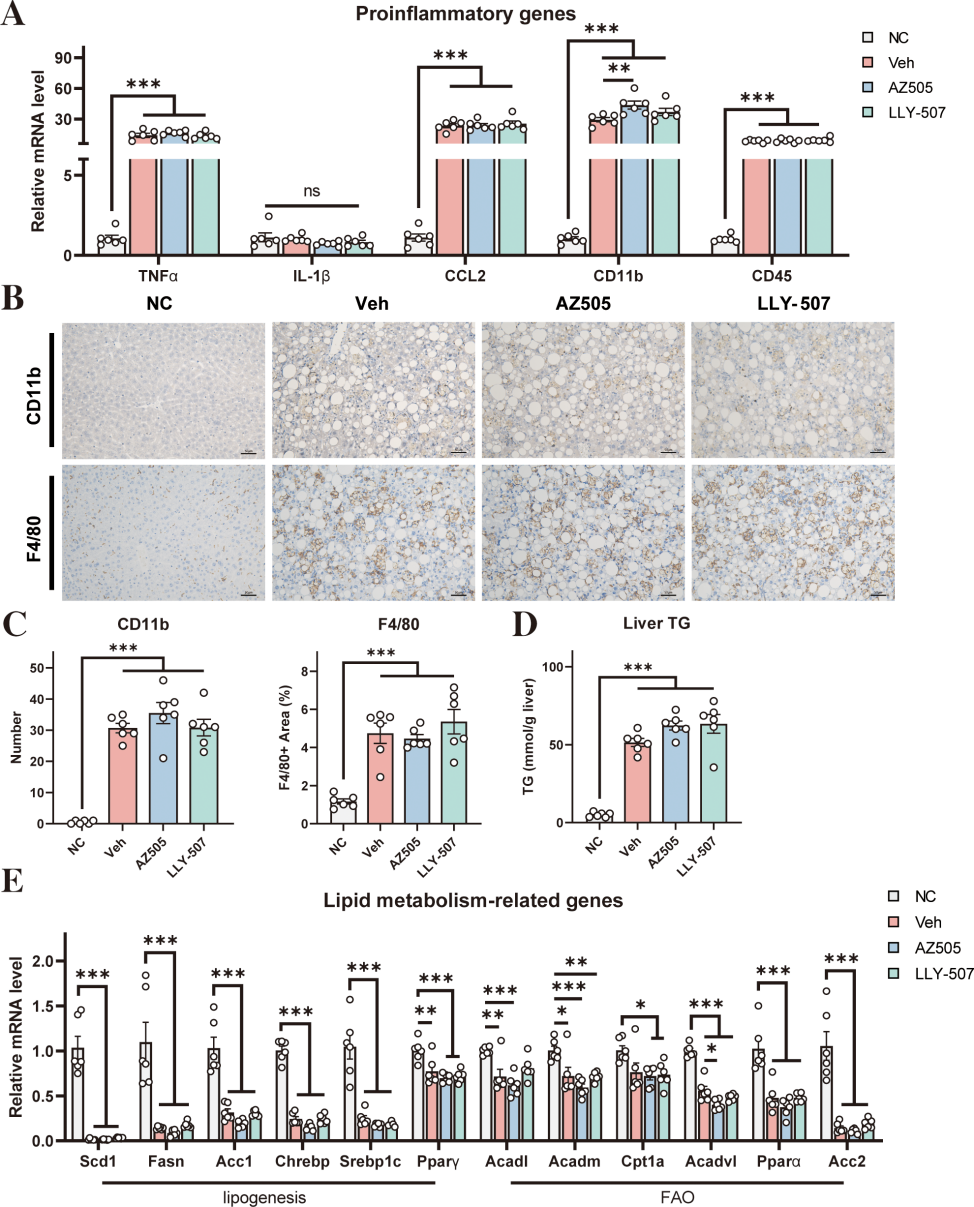
Effects of SMYD2 inhibitors on liver inflammation and steatosis in a mouse NASH model. **(A)** mRNA levels of pro-inflammatory genes in the liver (n = 6). **(B)** Representative immunostaining for CD11b and F4/80 in the liver sections of the mice (scale bars: 50 μm). **(C)** Quantification of the CD11b-positive cells and F4/80-positive area in the liver (n = 6). **(D)** Liver triglyceride level of the mice (n = 6). **(E)** The mRNA levels of representative genes involved in lipogenesis and FAO in the liver (n = 6). All statistical data are shown as mean ± SEM. *p < 0.05, **p < 0.01, ***p < 0.001, ns for non-significant.

NASH is always accompanied by the development of hepatic inflammation (28). We next assessed hepatic inflammation in our mouse NASH model. We analyzed the expression levels of a few representative inflammatory markers. Administration of CDAHFD induced robust expression of these inflammatory markers (**Figure 2A**). However, SMYD2 inhibitors could not lessen the CDAHFD-induced expression of these inflammatory markers (**Figure 2A**). Immunostaining for CD11b and F4/80 with liver sections indicated that the NASH diet could increase macrophage infiltration in the liver, while SMYD2 inhibitors had no obvious effect on improving it (**Figure 2B**). Quantification of the CD11b-positive cells and F4/80-positive area also revealed no significant changes between the drug-treated mice and NASH control mice (**Figure 2C**). These data, therefore, indicated that hepatic inflammation in NASH could not be improved by SMYD2 inhibitors AZ505 and LLY-507.

In addition, we analyzed the degree of steatosis of the liver. Administration of CDAHFD could significantly increase the triglyceride level in the liver (**Figure 2D**) indicating the development of hepatic steatosis by the NASH diet. However, the increased triglyceride level under NASH conditions could not be reversed by SMYD2 inhibitors (**Figure 2D**). We next analyzed the mRNA levels of a series of genes that control lipid synthesis and fatty acid oxidation (FAO) in the liver. In general, NASH mice had decreased expression of the genes involved in lipid synthesis and FAO (**Figure 2E**) indicating that hepatic steatosis in our NASH model is likely contributed by a decrease in FAO rather than an increase in lipid synthesis. However, both SMYD2 inhibitors AZ505 and LLY-507 could not alter the expression of these genes in the liver of the NASH mice (**Figure 2E**).

One possible limitation of our study is that we used CDAHFD diet in this study. CDAHFD is deficient in choline and methionine, and both of them are important for methylation reactions. SMYD2 is a methyltransferase, and its activity might be impaired without choline and methionine. Under this scenario, the effectiveness of SMYD2 inhibitors to alleviate NASH might be compromised. To avoid this potential problem, it is desirable to test other diets that can induce NASH in the future. Another issue that needs to be considered is the dose of the inhibitors used in the study. The dose of AZ505 at 10 mg/kg used in our study was identical to a previously reported dose (14). The dose of LLY-505 at 10 mg/kg was higher than what was reported (at 2 mg/kg) (29). It is therefore important to test other doses of the inhibitors in the future to fully elucidate the effects of SMYD2 inhibitors on NAFLD and NASH.

Overall, our results suggested that SMYD2 inhibitors AZ505 and LLY-507 could not improve NASH features induced by CDAHFD. Our results are different from Wang’s report in which it was found that hepatic steatosis was improved by AZ505 (14). In that study, they used Western diet to induce simple steatosis in the mice, not a NASH model (14). As Western diet mainly induces simple steatosis by upregulation of lipid synthesis, we hypothesize that SMYD2 inhibitors may improve simple steatosis by inhibiting lipid synthesis. However, SMYD2 inhibitors could not decrease fibrosis and inflammation of the liver in NASH mice. It is also worth noting that although CDAHFD is a well-characterized diet to induce NASH in mice (19–21), it will be imperative to assess whether or not SMYD2 inhibitors have any effect on other types of NAFLD or NASH models in the future. Nevertheless, findings from our study indicate that the potential of SMYD2 inhibitors as a novel therapeutic strategy for NASH warrants careful reevaluation. At least in murine models, the evidence supporting their pharmaceutical utility remains inconclusive and insufficient to establish their efficacy.

## Data Availability

The raw data supporting the conclusions of this article will be made available by the authors, without undue reservation.
